# The field of human building interaction for convergent research and innovation for intelligent built environments

**DOI:** 10.1038/s41598-022-25047-y

**Published:** 2022-12-21

**Authors:** Burcin Becerik-Gerber, Gale Lucas, Ashrant Aryal, Mohamad Awada, Mario Bergés, Sarah Billington, Olga Boric-Lubecke, Ali Ghahramani, Arsalan Heydarian, Christoph Höelscher, Farrokh Jazizadeh, Azam Khan, Jared Langevin, Ruying Liu, Frederick Marks, Matthew Louis Mauriello, Elizabeth Murnane, Haeyoung Noh, Marco Pritoni, Shawn Roll, Davide Schaumann, Mirmahdi Seyedrezaei, John E. Taylor, Jie Zhao, Runhe Zhu

**Affiliations:** 1 Sonny Astani Department of Civil and Environmental Engineering, University of Southern California, Los Angeles, USA; 2 Institute for Creative Technologies, University of Southern California, Los Angeles, USA; 3 Department of Construction Science, Texas A&M University, College Station, USA; 4 Department of Civil and Environmental Engineering, Carnegie Mellon University, Pittsburgh, USA; 5 Department of Civil and Environmental Engineering, Stanford University, Stanford, USA; 6 Department of Electrical and Computer Engineering, University of Hawaii at Manoa, Honolulu, USA; 7 Department of the Built Environment, National University of Singapore, Singapore, Singapore; 8 Department of Engineering Systems and Environment, Link Lab, University of Virginia, Charlottesville, USA; 9 Department of Humanities, Social and Political Sciences, ETH Zurich, Zurich, Switzerland; 10 Future Cities Laboratory Global, Singapore ETH Centre, Singapore, Singapore; 11 Department of Civil and Environmental Engineering, Virginia Tech, Blacksburg, USA; 12 Trax.Co, Toronto, Canada; 13 University of Toronto, Toronto, Canada; 14 Lawrence Berkeley National Laboratory, Berkeley, USA; 15 Salk Institute for Biological Studies, La Jolla, USA; 16 Department of Computer and Information Sciences, University of Delaware, Newark, USA; 17 Thayer School of Engineering, Dartmouth College, Hanover, USA; 18 Building Technology and Urban Systems Division, Lawrence Berkeley National Laboratory, Berkeley, USA; 19 Chan Division of Occupational Science and Occupational Therapy, University of Southern California, Los Angeles, USA; 20 Faculty of Architecture and Town Planning, Technion – Israel Institute of Technology, Haifa, Israel; 21 School of Civil and Environmental Engineering, Georgia Institute of Technology, Atlanta, USA; 22 Delos Labs, Delos, USA; 23 Weitzman School of Design, University of Pennsylvania, Philadelphia, USA

**Keywords:** Civil engineering, Quality of life, Environmental impact, Mechanical engineering, Occupational health

## Abstract

Human-Building Interaction (HBI) is a convergent field that represents the growing complexities of the dynamic interplay between human experience and intelligence within built environments. This paper provides core definitions, research dimensions, and an overall vision for the future of HBI as developed through consensus among 25 interdisciplinary experts in a series of facilitated workshops. Three primary areas contribute to and require attention in HBI research: humans (human experiences, performance, and well-being), buildings (building design and operations), and technologies (sensing, inference, and awareness). Three critical interdisciplinary research domains intersect these areas: control systems and decision making, trust and collaboration, and modeling and simulation. Finally, at the core, it is vital for HBI research to center on and support equity, privacy, and sustainability. Compelling research questions are posed for each primary area, research domain, and core principle. State-of-the-art methods used in HBI studies are discussed, and examples of original research are offered to illustrate opportunities for the advancement of HBI research.

## Introduction

Technology is rapidly advancing and, in doing so, is changing not only virtual realms but also our everyday physical environments. Smart connected devices and advancements in sensing, actuation, and communication are converging to bring new modes of interaction and experience within our built environment. This transformation is leading our society towards a different way of engaging with the spaces in which we live, work, play, and learn. Built environments of all shapes, sizes, and manifestations are becoming intelligent partners that handle operational and repetitive tasks to support basic human needs, promote human health and well-being, and facilitate creative, intellectual, social, and emotional pursuits. Human-Building Interaction (HBI) represents the next frontier in convergent research and innovation to enable the dynamic interplay of humans and intelligence within a built environment. In this context, we define a building’s intelligence as the overall awareness and respect for the needs and preferences of humans and other stakeholders combined with the capabilities necessary to adapt and respond in an environmentally conscious manner that enhances human health, well-being, and performance. The HBI field studies how humans perceive, interact with, navigate through, and spend time within built environments and how reciprocating actions of humans and buildings can positively influence each other’s behavior.

In this paper, we provide a vision for the convergent field of HBI, intending to formalize critical principles, methods, and outcomes. This paper builds on prior efforts to characterize the field. Many authors have attempted to define HBI through the lens of Human–Computer Interaction (HCI)^1–3^ or by focusing solely on the temporal constraints of HBI^4^. In a more concerted effort^5^, researchers in human factors engineering, HCI, and architecture developed definitions, identified research directions, and established sub-agendas meant to guide the work of the individual disciplines^6^ toward a shared mission and scope of work for HBI^7^. We were inspired to expand on this previous work by establishing an even broader perspective that can promote both individual disciplinary efforts and meaningful interdisciplinary research to advance effective HBI. This broad group of experts not only included engineering, human factors, architecture, and computer scientists (within HCI and beyond) but also involved perspectives from social, behavioral, health, cognitive and occupational scientists. Together, we provide a shared mission and vision for HBI by establishing universal terminology, defining core dimensions, identifying key research questions, and outlining approaches and methods. Using a broad interdisciplinary perspective to shape the convergent field of HBI can result in a more significant impact by cultivating collaboration and coordination among the scientific community that closes gaps in current disciplinary-specific research. This shared language and collective vision set the groundwork for valuable disciplinary, interdisciplinary, and transdisciplinary advancements in HBI that contribute to an improved fundamental understanding of and applications that maximize how intelligent built environments interact with, adapt to, and support humans.

### Consensus methods

A diverse group of 25 industry-based and academic scholars from various disciplines convened in a writing workshop meant to minimize isolation (which can be an issue in interdisciplinary fields) and maximize cooperation (eliminating duplicate efforts across different domains) among individuals interested in HBI. Research fields represented in the workshops included: built environment design, construction, and operations (including architecture and various engineering disciplines such as civil, mechanical, electrical, environmental, and industrial engineering), energy, transportation, applied sciences, computer science, artificial intelligence and computer vision, communication and textual studies, information and communication technologies, human factors, HCI, human-centered design (HCD), humanities and social sciences, cognitive science, psychology, medical informatics, public health, environmental and occupational health, occupational science, orthopedics, and rehabilitation.

The workshop was composed of four professionally facilitated online sessions, each 4-h long, held biweekly from January 2022 to March 2022. Before and during the sessions, participants’ perspectives and ideas were solicited using virtual “sticky notes” in the workshop-facilitation platform^8^ related to the definition of HBI, the vision and scope of HBI, the research areas of HBI, and the development of this paper, including sections, title, and appropriate publication venues. Participants interacted in several ways to share their perspectives, ideas, and concerns, including verbal discussions in plenary sessions with all participants, oral discussions during breakout sessions, post-hoc online meetings, and through Slack channels or email messages. Participants voted on the various outputs generated by the group to identify, prioritize, and come to a consensus on each component of this paper. All participants contributed to writing, editing, or commenting on this paper, primarily occurring asynchronously between and after the four workshop sessions. Participants contributed content specific to their expertise and reviewed all content to ensure that the perspectives of their disciplines were represented throughout.

In the following sections, we present the consensus outcomes of this HBI workshop, including a shared interdisciplinary definition, vision, and impact for the field; descriptions of the overarching areas and associated transversal dimensions of HBI research; and examples of research studies and methods within HBI. All experimental protocols were approved by the respective universities’ ethic committees (University of Southern California, Carnegie Mellon University, Rensselaer Polytechnic Institute, Technion—Israel Institute of Technology). All methods were carried out in accordance with relevant guidelines and regulations and informed consents were obtained from all participants.

## HBI definition

**Figure 1:**
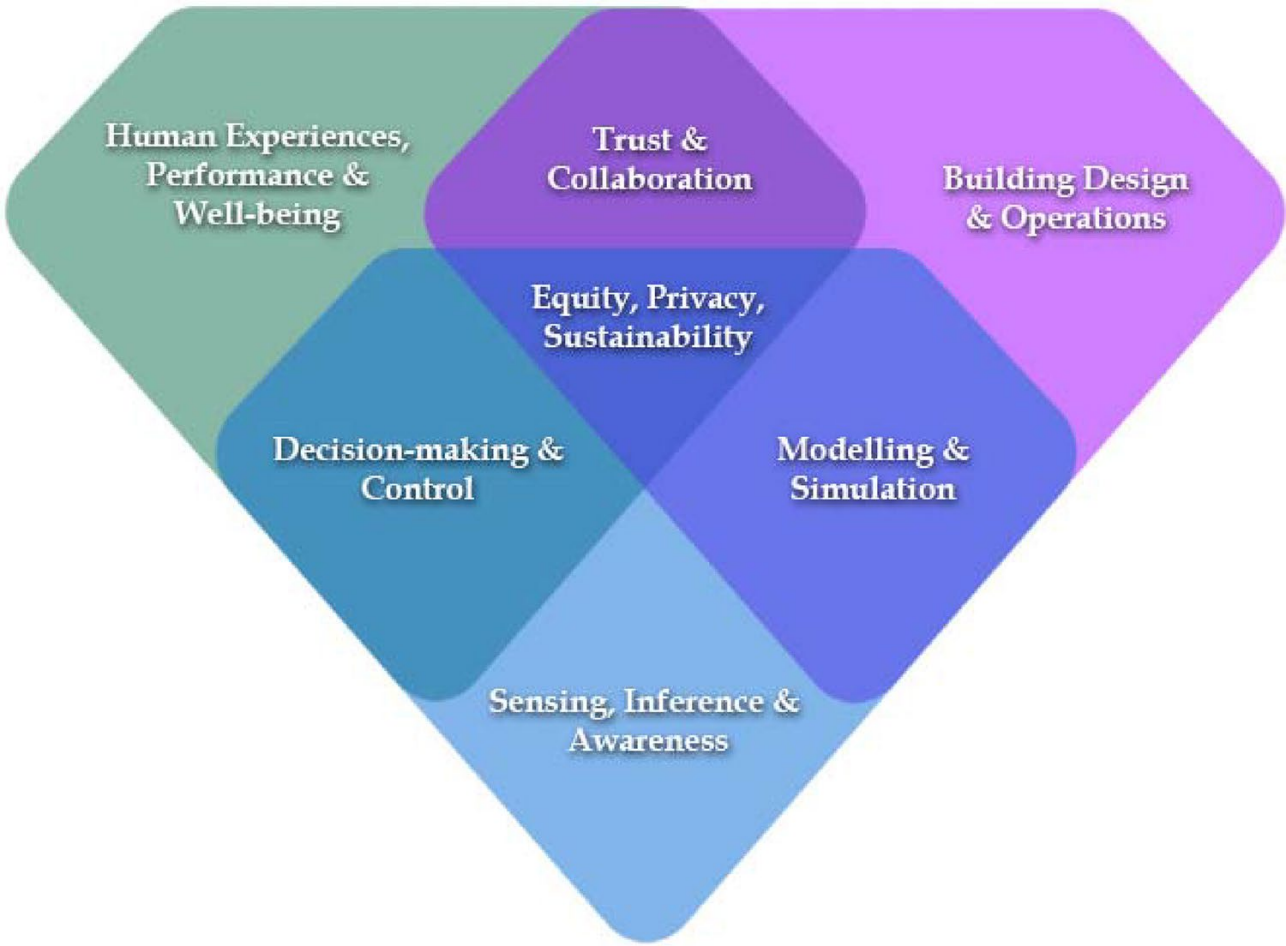
Primary HBI research areas, intersecting interdisciplinary research domains, and core principles of all HBI research. Image credit: Basma Altaf.

Human-Building Interaction (HBI) is an interdisciplinary field that aims to understand how built environments affect human outcomes and experiences and how humans interact with, adapt to, and affect the built environment and its systems. At its core, HBI focuses on enabling built environments that can learn, adapt, and evolve at different scales (individual building, community level, city level) to improve the quality of life of its users while optimizing resource usage and service availability. As a result, HBI researchers and practitioners explore the mutual impact between buildings and humans, observe how users interact with built environments, and design technologies to support novel interactions in such spaces. Figure 1 shows the critical elements of this definition.

Within HBI, we describe buildings as built environments created by humans to service our needs, such as to ensure safety and comfort, improve the quality of life, and accomplish personal goals (e.g., work, play, relax, sleep) and societal-driven goals (e.g., reducing energy consumption, recycling). The considered built environment may refer to a single building or numerous, interconnected, physically adjacent buildings that might create a community, campus, city, or network of buildings. Here, we focus on buildings and their immediate external surroundings but acknowledge that some work in HBI is more expansive in scopes, such as smart cities and infrastructure. We also consider buildings to be complex systems that consist of, among other things, the building envelope, heating, ventilation, and air conditioning (HVAC) systems, electrical systems, energy generation and management, water and plumbing, lighting and daylighting, intelligent controls, entertainment, interior design, furniture, and modes of movement in, out, and around the building. These sub-systems actively and passively interact with humans and, as a result, impact human experiences, interactions, activities, and other outcomes.

Interactions among humans and the built environment range from passive to active, with various direct and indirect interfaces. Passive interactions affecting human users include adjustments to the building system’s operations to accommodate the needs of the humans or minimize impacts on the environment. For example, HVAC systems regulate the indoor environment's temperature, humidity, and airflow, impacting a human’s thermal experience. Lighting/shading systems adjust light color and intensity, which could affect humans’ physical and psychological well-being due to their impact on circadian rhythms^9^. Similarly, spatial configuration and indoor environments are often designed to meet the needs of activity, mobility, or other participation that can affect building operation systems. For example, open-plan workplaces can encourage collaboration while walled offices and meeting rooms provide privacy. Yet, each of these configurations can lead to inefficient use of space or ineffectual operations by building systems. On the other hand, active interaction includes human action to change building systems or the built environment. Operating windows and controlling heating setpoints via an interface represent well-known forms of active interaction with building systems. Smoking cigarettes and cooking represent indirect but active interaction with the built environment that can, in turn, impact human outcomes and experiences and trigger reactions from building systems, such as safety sensors triggering an alarm when detecting smoke. In the context of HBI, such passive and active interactions are facilitated through intelligent interfaces^10^ and sensing^11^ and the building becomes aware of human attitudes, needs, habits, norms, motivations^12^ and chooses to learn, adapt, and evolve for best human outcomes and experiences^13–15^.

Individual and collective human interactions with buildings can take many dynamic forms across the passive-active interaction spectrum. Humans and buildings can interact with one another through new and adaptive multimodal interfaces enriched by sensing and computing^16, 17^, including the use of motor functions, such as speech, gesture, and navigation^18^. Yet, these interactions are inherently different than the ones in the field of HCI, where there is a defined mode of interaction (a computer), often through hardware explicitly created for the interactive purpose. In buildings, this interaction is facilitated through embodied and built-in intelligence within building elements^2, 5^ that may not be initially designed for intelligent interaction, as well as other technologies added to the environment (e.g., windows, doors, building furniture like desks or chairs, light switches, thermostats, smart paint^19, 20^). Not only is the significant range of available features relevant to HBI widely varied in design, but these features are also typically multifaceted in function and application. For example, interactions can target many physical and sensory outcomes (e.g., thermal, and visual comfort^21–25^, satisfaction^26–32^, health^33^), and one person’s interaction with a building can easily influence many others^34^.

## HBI vision

We envision that the field of HBI will enable a future where widespread synergistic relationships between humans and built environments exist to support individuals and advance broader societal goals. This vision involves the study of humans not solely as occupants, end-users, or homogenous actors within a built space but as individuals with unique lived experiences, personalities, capabilities, and goals. HBI research aims to support positive engagements as these diverse individuals participate in activities within and around the built environments in which they live, work, and play. By understanding human attitudes, motivations, habits, and norms and accounting for occupants' safety, security, and privacy, HBI seeks to engage buildings with occupants to produce better personal and societal outcomes. Similarly, HBI involves studying built environments not only as physical spaces and operational systems but as the foundation for technologies that can sense and infer the needs of, provide support to, and interact with humans. As such, the field of HBI is interested in enhancing both new and existing physical spaces and being deeply engaged in supporting the future of intelligent built environments.

## HBI research dimensions

As the foundation, HBI scholarship merges three primary research areas related to humans, buildings, and technology, each rooted in the scholarship of individual disciplines across social, behavioral, health, building, and computer sciences: *Human Experiences, Performance, and Well-being*; *Building Design and Operations*; and *Sensing, Inference, and Awareness.* Intersecting interests between pairs of these research areas give rise to three interdisciplinary research domains: *Trust and Collaboration*, *Decision-making and Control*, and *Modeling and Simulation*. At the center of all HBI research are core principles such as *Equity, Privacy, and Sustainability* that should be incorporated or considered in every aspect of HBI scholarship. In this section, we provide descriptions of these research dimensions to establish a shared language for the interdisciplinary field of HBI.

### Primary research areas in HBI

#### Human experiences, performance, and well-being

Research on technology integration into buildings has focused chiefly on top-down technology developments that largely neglect the dynamics of different building users with diverse preferences and needs^35^. Human aspects of HBI research should consider the impact of an environment and its constituent elements on human’s physiological and psychological experiences, activities, and performance across a spectrum of short and long-term horizons. Most of our daily activities take place inside buildings used as homes, workplaces, schools, retail stores, healthcare facilities, and other social or business spaces. Significant opportunities exist to employ HBI approaches that enable a seamless connection between users and buildings to support positive human experiences. Buildings that produce an ongoing sense of fulfillment^5, 36^, even during mundane activities^37^, will require an ambitious human-centered focus to define the goals of engineered systems that promote a wide variety of human experiences, such as comfort, satisfaction, convenience, health, well-being, safety, lifelong learning, communication, social engagement, and productivity. Within our HBI infrastructure, we imagine a future in which buildings become perceptual and cognitive environments, encompassing both users and the physical infrastructure that shapes and supports human intent, perception, and behavior. In such a cognitive environment, humans are integral to the system rather than a variable to work around^38–41^.

There are significant opportunities for the built environment to affect humans and for humans to impact built environments, represented by research focused on human experiences and responses. Such studies have quantified and modeled the impact of comfort on human satisfaction and performance^26–32^ or have investigated contributors to the physical, mental, social, and spiritual health and well-being of building occupants^33^. In addition to examining occupant or building-focused outcomes, HBI research can also investigate complex intersections between human behavior and these individual or shared outcomes. In a well-studied HBI example problem, behavioral scientists have pointed to an energy efficiency gap between the design intent of technologies and their realized performance in actual built environments^42^, primarily due to human behavior. Research in this area investigated, among others, the drivers of human behavior (e.g., norms, attitudes)^12^, social practices that impact energy use^43–45^ and interventions to produce behavioral change^46–48^. This example highlights the importance of understanding the complex intersections among diverse, individualized human experiences and human behavior throughout daily activities as these complexities inform, shape, and influence the built environment.

Interdisciplinary collaboration among social, health, and behavioral scientists with architects, engineers, and computer scientists is critical to advancing the theoretical and practical impacts of HBI research^49^. A primary example of such collaborations is the study of energy feedback^50^ and user interfaces for energy devices^51^, which stemmed from the interface between social science and HCI. These interdisciplinary collaborations are further facilitated by developing more granular and human-centric sensing technologies. Technologies that can identify occupant activities, emotional states, and needs can be used by intelligent built environments to support engagement, well-being, and performance. Moreover, these technologies can provide the means for a synergistic or cooperative approach between the building and occupants to influence synergistic behaviors by both sides that can achieve individual and shared goals. It is vital that future research leverage this synergistic perspective to understand how humans and building technologies ascribe shared value. Synergistic approaches are critical as AI begins to automate tasks previously under the occupant's control (e.g., opening blinds, preparing coffee, updating thermostat schedules). Although such actions by the built environments are often meant to support the user or address a broader human-defined objective (e.g., reducing energy), some humans may value personal control over other benefits^49, 52^. As such, interdisciplinary approaches that investigate the distributed interaction and agency across all the human users and building technologies are vital.

#### Building design and operations

Whether it is designed to be a smart built environment from the outset or may be augmented/retrofitted to include technology aspects, the physical building and its operations are critical components of HBI. Disciplinary knowledge within architecture and various engineering fields such as mechanical engineering, electrical engineering, architectural engineering, civil engineering, and building science provide a strong foundation for HBI research. Research on building processes and practices over the last decade has led to innovations in design methods^53–55^, engineering systems such as HVAC equipment and appliances^56, 57^, lighting^58, 59^, building materials like glazing systems^60, 61^, construction processes^62, 63^, and building management and controls^64, 65^. In HBI, it is important to move beyond these building-centric foci to leverage sensed awareness of the needs and behaviors of their occupants so that user experiences and outcomes are optimized against often-competing external variables such as energy availability, extreme events (e.g., wildfires and public health concerns), weather events, schedules, or the needs of other users.

Incorporating social, behavioral, health, and computer scientists in HBI research will ensure that the dynamic behavior of building occupants is accounted for in the design process and day-to-day operations of physical buildings. Human-centered design approaches enable the creation of environments that support evolving needs of their occupants. One such approach is cognitive-grounded design, which is based on human perception and cognition research. Using this approach, one study examined how people process and respond to spatial and social stimuli to inform design decisions related to wayfinding scenarios^66^. Another example is biophilic design, which includes the design of spaces that reflect the innate affinity humans have for nature to support human well-being that incorporates considerations such as access to natural light, views of nature, and use of natural materials^67^. Similar approaches in HBI research could explore how to best connect built environments to communities, cultures, and contexts, including considerations for designing the space where the building meets the external urban or rural area^68^.

From an operations perspective, AI-driven adaptations of built environments include changes in building operations or features in response to occupants’ short- or long-term needs. These operations include physical environmental components such as adequate lighting, optimal indoor air quality, and thermal comfort, as well as human performance considerations such as promoting social engagement, productivity, and safety^13^. In the field of HBI, building operations should consider incorporating technology and human performance in day-to-day resource management, such as for the improvement of HVAC and other physical building operations, as well as improving wayfinding activities to manage efficiency in human mobility within busy environments^18^. It is also vital that HBI research consider support for building operations for extreme scenario responses such as fire accidents^69^ or violent attacks^70^.

#### Sensing, inference, and awareness

Technology aspects of HBI research incorporate the primary areas of sensing, inference, and awareness that underpin actuation, communication, and other interfaces with human and building components of the HBI research infrastructure. Interdisciplinary research in these areas falls at the intersection of computer science and other areas of computing and engineering, including civil engineering, mechanical engineering, electrical engineering, and industrial and system engineering.

Sensing technologies include various physical environmental devices, personal or portable wearable devices, and sensor deployment strategies. HBI research has examined numerous environmental sensors that can quantify human activities, experiences, and performance, and monitor changes within the physical or social environment^16, 71–73^. Internet of Things (IoT) provides a way to connect building operations such as security and access control, predictive maintenance, structural health, fire detection and so on^74^. Similarly, wearable sensors that measure physiological variables, such as skin temperature, perspiration rate, and heart rate, can assist in understanding human health and well-being related to engagement within built environments^31, 75^. These methods rely on sensing technologies that provide robust data throughout daily activities that require the direct engagement of the human or building. On the other hand, non-intrusive technologies including infrared thermography^32, 76^, optical imaging^30, 77, 78^, ambient sensing^17^, and real-time visual perception using human eye pupil size measurements^79^ have been proposed as alternative non-contact methods to evaluate human experience within built environments.

Regardless of the sensing method, data from these systems can be used to infer higher-level knowledge, such as recognizing human presence, activities, and interactions, as has been done in activity recognition research^80–86^. Reasoning out broader constructs can be achieved through direct inference from data gathered by the sensing hardware or through virtual sensing supported by inference algorithms^87^. Using these methods, intelligent buildings can develop, calibrate, and refine models of human occupants to infer the emotional state, physical abilities, or social dynamics of individual occupants or groups to create opportunities for buildings to interact with people more appropriately. For example, if an intelligent building infers that someone is operating a wheelchair, it could recommend alternative paths that accommodate their needs. In an emergency, when inferring relationships, such as a mother holding the hands of young children, a more appropriate egress path may be presented to the mother that accommodates the children’s abilities. Alternatively, a building could infer the emotional state of its occupants and operate the systems accordingly.

When captured over time or across a network of buildings, sensing and related inference data can be aggregated to develop activity patterns, thus giving the building occupant-awareness. Broad awareness of human needs, environmental needs, or other aspects of social context can allow the building to leverage predictive functions to maximize support required in the environment while minimizing disruptions to building operations or human engagement. Given this awareness, when an intelligent environment is aware that it currently is not supporting and cannot fully support the needs of occupants, it can recommend optimal changes or improvements to the owner/operator of the building to better meet social needs and environmental goals.

#### Overarching research opportunities

Current studies mainly consider built environments as static containers rather than proactive partners. New HBI research opportunities involve analyzing the collaborative interactions between humans and intelligent buildings equipped with sensing, inferring, and communication abilities. More human-aware and cognizant building design and control systems could inform dynamic building adaptations in response to and anticipation of people’s emerging needs^14^. For example, intelligent environments equipped with sensing and awareness technologies could potentially consider the dynamic state of the building and its occupants at a given time to recommend optimal resource allocation scenarios, including spaces, people, equipment, and energy services. Research can support such involvement in day-to-day operations, in response to unfolding situations, and in predicting and preventing undesired situations. These approaches can simultaneously improve human engagement while considering the building’s impact on its occupants and broader impact on the natural environment. Table 1 summarizes research needs across these three primary research areas in the form of sample research questions.

**Table 1:** Sample research questions related to human, building, and technology aspects of HBI.

**Human experiences, performance, and well-being**
How can built environments augment user performance (such as productivity or safety)?
What are the effects of HBI on joint human-automation performance?
How do built environments’ design and operations passively and/or actively impact occupants’ health and well-being?
How can interfaces in the built environment actively “nudge” occupants towards adopting changes for healthier behavior, and how can “nudging” be converted to active awareness and participation in positive actions among the occupants?
How can psychological, social, and economic theories help building systems and AI to enhance occupant experience, performance, and well-being?
What role does AI play in addition to humans in making decisions about building operations (e.g., should the AI system be in charge based on predetermined human requirements or should the human be in charge based on AI suggestions?)
How might built environments help humans, families, and organizations achieve their goals?
**Building design and operations**
What is the difference between designing non-intelligent buildings vs. intelligent ones that are aware and proactively cater to the needs of their occupants?
What approaches could be used to perform cognitive-grounded analyses of the collaborative interactions between humans and intelligent buildings equipped with sensing, inferring, and communication abilities?
How might we design spaces that are intuitive to navigate for humans with different physical and cognitive abilities both in the day-to-day and emergency scenarios (e.g., to help their occupants to shelter during an active shooter incident)?
How could natural elements be seamlessly integrated into the building design process to enhance the well-being of different populations over time?
How can AI-driven adaptations of built features be programmed to respond to and anticipate occupants’ short- or long-term needs?
What are the opportunities and challenges for incorporating automated operational adaptations in existing buildings vs. building intelligent adaptation methods into new designs?
To what degree does enabling occupant awareness and interactivity in built environments promote low-energy and low-carbon building operations?
**Sensing, inference, and awareness**
What are optimal modalities and configurations of sensors that result in high accuracy and robust data collection and inference in a privacy-preserving and unbiased manner?
What sensor modalities are needed to be added to the mix of Internet of Things (IoT) devices to facilitate more ambitious goals in the HBI field?
Given the combination of fixed and dynamic sensors within the built environment, with a varying set of IoT devices used by occupants, how can a sensor fusion system best normalize the data features to support higher-level functionality such as activity recognition?
How could buildings infer and quantify each specific occupant’s habits, experiences, and social interactions through the emerging sensing technologies?
How could a general graph of human activities be generated and populated given myriads of sensor fusion results from various building types? How could the social, temporal, and semi-hierarchical nature of the relations between human activities be represented?
What representations could be used to develop an awareness of human needs, social contexts, and environmental needs so that levels of compliance to those goals can be estimated? How can recommender systems be developed to automate the suggestion of optimal modifications to increase compliance?

### Interdisciplinary research domains in HBI

Three interdisciplinary research domains sit at the intersections of the primary research areas in HBI (see Fig. 1). Trust and collaboration at the intersection of human and building aspects of HBI include human-building teaming, synergistic human behavior and building operation changes, and other actions that require trust and collaboration to support shared goals between the building and its occupants. The domain of decision-making and control includes topics at the intersection of the technology and human aspects of HBI, such as occupant-centric controls, context-aware operations, intelligent automation, bidirectional adaptation, artificial intelligence, machine learning, and data analytics. Finally, the interdisciplinary research domain of modeling and simulation includes topics at the intersection of the building and technology aspects of HBI, such as physical and digital system adaptations, design intent, design intervention, computational modeling, and predictive maintenance. Table 2 summarizes research needs pertaining to these interdisciplinary research domains within HBI.

**Table 2:** Sample research questions related to interdisciplinary research areas of HBI.

**Trust and collaboration**
How might we better design novel interactions that increase trust and collaboration between built environments and their users?
How will these interactions change the behavior of humans and built environments?
How do, and what type of, interactions with building structures, systems, and operations influence how humans perceive, experience, and use spaces?
What types of user interfaces and modes of communication promote effective collaboration and trust to advance shared goals between the building and human occupants?
How does the level of automation affect trust in technology?
How does trust in technology change based on users' tasks in built environments?
How will novel HBIs change people’s expectations of the built environment?
How will the type of collaboration affect the way that humans use the built environments?
**Decision-making and control**
How will control systems respond to a diversity of human needs?
Should a bottom-up approach (intelligence growing from individual smart devices) or a top-down approach (a smart central building manager) be taken? Or is it this goal dependent, and for some goals, the system should consider a top-down approach, and for other goals, a bottom-up approach might be a better option
How do users react to intelligent and aware spaces or building (e.g., buildings that dynamically adjust the color temperature according to user activities or building geometry support users)?
How could smart systems be developed to be scalable and transferable?
**Modeling and simulation**
How can we generalize and incorporate well-being considerations into design tools (e.g., Building Information Modeling)?
Which physical and digital adaptations are most effective for specific populations/cultures/building styles?
How can the physical layout be designed to reflect the organizational structure, levels of sharing, and visibility (physically and conceptually)?
What are the tradeoffs between physical materials and digital representations?
How can computational models of human behavior account for the building occupants' cultural, physical, and psychological traits?
How can generative design approaches account for designers’ tacit knowledge?
How can Machine Learning (ML) and Deep Learning (DL) approaches replace or integrate traditional Multi Agent Systems (MAS), which often require long computational times?
What is the role of digital twins in HBI research? How can future building digital twins preserve building occupant privacy?

#### Trust and collaboration

When the built environments become infused with intelligence, there will be new opportunities for human-building teamwork that calls for collaboration and trust. Prior interdisciplinary work has considered factors that encourage trust in and collaboration with automation for various tasks (e.g., in manufacturing, aviation^88, 89^). There is a developing empirical body of research for trust in automation for intelligent built environments, which requires collaborations among many disciplines, including architecture, cognitive science, engineering, computer science, and human factors and industrial-organizational psychology experts. With more intelligent buildings^1^, there will be an increased level of automation based on the type and function of the environment^88^. In addition, new modes and types of interaction will be needed to fulfill the goals of both humans and the built environment. For built environments to develop the intelligence required for collaboration, users need to trust the environment with data acquisition, processing, storage, and decision-making. Ambitious HBI goals also require user trust in accepting the actions, decisions, and recommendations from the built environments’ features. Research on trust could be built on similar research efforts involving the privacy and security of intelligent systems and environments, resulting in a better understanding of how users and communities perceive the role and functionalities of such systems. Additionally, it is necessary to consider the implications of trust based on how the sensing, inference, and awareness solutions support goals and objectives that align with those of different stakeholders. For example, the goals of building occupants may be counter to those of building managers/owners, who could lead reduce trust among various stakeholders. Finally, building design and operations might have an impact on trust and collaboration. According to the literature, layout design and nature stimuli can provide a sense of trust for occupants. For instance, physical proximity promotes collaboration, bonding and trust^89^, and trust bond among residents of a place can be associated with the sense of satisfaction about sharing environments^90^. In this regard, open-plan office environments with assigned work spaces help participants foster trust and respect among each other^91^. On the other hand, shared work environments, and in particular hot-desking, are associated with distrust in work environments^92^. As another example in regard to the biophilic design of buildings, exposure to more beautiful images of nature led participants to be more generous and trusting in comparison to exposure to less beautiful images of nature^93^.

#### Decision-making and control

As a classical HBI framework, occupant-centered controls in buildings aim at improving occupant satisfaction and perceived service quality (e.g., thermal and visual comfort) while accounting for sustainable practices (e.g., minimizing energy consumption^94^ or integration of renewable resources^68^). A wide range of approaches have been explored, from simple presence-responsive systems (e.g., lighting triggered by an occupancy sensor) to complex predictive techniques (e.g., model predictive controls) based on human activity and preferences^95^. Challenges remain in the development of decision-making and control models that can identify user routines and patterns, predict shifting or dynamic behaviors, and infer preferences to support user-adaptive building operations. Interdisciplinary HBI research is necessary to identify more granular and accurate inference across routine and dynamic human behaviors, diverse human preferences, and various emotional and physical states across all human occupants. Improved inference, informed by algorithmic frameworks and enabled by new sensing modalities, will lead to better decision-making and control to optimize building operations, improve the experiences among diverse users within individual buildings, and balance such needs against external variables. Building-level decision-making, control, and awareness can provide a foundation for broader system-level operations, such as interconnections among buildings and the surrounding neighborhoods across communities or within more extensive infrastructures. To this end, future interdisciplinary HBI research is necessary to identify human-centered AI solutions that are scalable, sample efficient, and safe. It will be essential to develop studies of building-level intelligent agents that are informed not only by local objectives and constraints but also by the intelligent system's role in larger-scale coordination. System-level operations might include community-based energy management for demand-response and renewable energy integrations or smart building-city coordination to inform and enhance first responders’ response to emergencies.

#### Modeling and simulation

Architects and engineers have long used digital representation tools to model building features, evaluate features relative to design intentions, and enhance communication across design stakeholders^96^ such as Building Information Modeling (BIM)^97^. BIM, however, lacks information about the behaviors of building occupants as well as their interactions with the built environment^98^. Since these aspects are at the core of HBI, it is thus imperative to extend existing modeling tools and practices with this new emphasis. To this end, a new research area involves the computational simulation of human behavior in buildings using multi-agent systems (MAS). In MAS, prospective building occupants are modeled as agents that interact among them as well as with the surrounding built environments to goals related to the type of organization that occupies the building^99^. These approaches have been applied to model operational performance in process-driven facilities like hospitals^100^, wayfinding in multi-level buildings^101^, occupancy patterns in university campuses^102^, thermal and acoustic comfort in office spaces^103^, and emergency evacuation scenarios^104^. Generative design approaches also show promise in coupling human-centered evaluation metrics with parametric models of buildings to automatically generate, sort through, and evaluate several design options and recommend the ones that balance tradeoffs between building and human performance metrics^105^. This approach requires interdisciplinary research efforts that bring together architects, engineers, social scientists and building scientists. BIM models have also been coupled with real-time sensing technologies to dynamically update a digital representation (often called a Digital Twin) of a building or its parts/systems^106^. Digital twins of the built environment provide a foundation for real-time evaluation of building systems and data-driven forecasting of future performance. Computational simulation of building performance could also be incorporated into digital twins to inform real-time decision-making in day-to-day and emergency scenarios. Interdisciplinary HBI research could use the existing approaches as a starting point to develop more comprehensive tools and techniques that better inform the design and operational adaptations at different time scales while also accounting for more detailed occupant profiles, which include cultural, geographical, psychological, physical, and economic traits, as well as passive and active interactions with intelligent control systems.

### Core principles in HBI

Leveraging interdisciplinary perspectives, the field of HBI emphasizes greater understanding and enabling of positive two-way interactions between humans and built environments that include passive solutions while promoting active engagements. By supporting bi-directional human-building synergies and promoting responsible innovation (similar to calls in HCI^107^), successful HBI efforts can result in meaningful changes to the design, operation, use, and assessment of built environments that have profound effects on our lives, our society, and our environment. Among many areas of broad impact, thoughtful HBI research should be conducted with consideration of core principles that reflect contemporary societal goals: (1) promoting equity and inclusion of individuals who engage within and around buildings, (2) addressing evolving concerns of privacy and security related to the increased use of technologies within buildings, and (3) supporting sustainability and resilience in the face of environmental, social, or other hardships (e.g., disaster response, homelessness). Table 3 summarizes the research questions pertaining to the core principles of HBI.

**Table 3:** Sample research questions related to core principles of HBI.

**Equity and inclusion**
How could universal and inclusive design concepts be integrated with the theoretical disciplines and practical applications of HBI?
How can the dynamicity of interactions between occupants and buildings enable us to consider the specific needs of some groups of societies that are more susceptible to features of physical environments?
How can we include and adapt to the needs of different groups in the design and implementation of HBI-based solutions?
How can we rethink making low-cost HBI solutions to extend the usability of these solutions among the public?
**Privacy and security**
What new security and privacy concerns do device-human-building interactions raise?
Can we develop new adaptive and flexible control schemes to secure an expanded dynamic, diverse, and heterogeneous environment with good user experience and application performance?
How do we address the issues raised by more sub-systems being added into buildings or conflicts between different application requirements?
What are the tradeoffs between security/privacy and application performance or user experiences?
How can building systems respond to personalized privacy profiles?
**Sustainability and resilience**
How might we balance conflicting objectives and tradeoffs of comfort, well-being, energy and CO_2_ reductions, and resilience?
How do we move beyond individual buildings and coordinate buildings at a neighborhoods or city scale?
How do we plan for the inevitable need to renovate, retrofit, and retire buildings?
How could HBI technologies and solutions lead to increased resilience and operational flexibility of buildings and communities?
How could HBI research assist with resilience towards extreme events due to climate change such as wildfires, heat waves, flooding, etc.?

#### Equity and inclusion

HBI research must consider diversity across the many types of individuals who occupy or use buildings to promote accessibility, inclusion, and equity in applying technological supports. There is a long-standing underrepresentation and consideration of user diversity in building-related research that includes many marginalized groups and minorities^108, 109^. In the study and development of HBI, there is a need to understand differences in engagement and support required across different genders, age ranges (i.e., including children, youth, and older adults), and socioeconomic statuses^110^. Moreover, HBI solutions and technologies should determine how support is equitable and inclusive for individuals from communities of color and people with varied physical abilities or mental health conditions^111, 112^. Finally, there is an opportunity to expand HBI efforts to support the needs of displaced groups, migrants, and unsheltered individuals^113^. HBI professionals can lead standards that encourage inclusive behavior and recognize ongoing exclusions and societal biases to ensure that diverse populations are represented in user studies and in testbed or real-world deployments. Applying a diversity lens within research practices is a significant consideration to avoid bias inherent to humans, which can be inadvertently carried forward into the resulting technology^109^. Universal and accessible design is necessary to support an inclusive culture and is key to ensuring that built environments support diverse human needs and effective activity engagement^108^. HBI technology has the potential to maximize the adaptability of design for individuals with differing physical, social, and sensory abilities^114^. Although many HBI innovations will be technologically advanced, HBI efforts should also consider applications for economically disadvantaged regions and low-tech spaces. In fact, because low-tech or no-tech built environments constitute most of the current building stock, the extrapolation and adaptation of innovations from high-tech built environments is an opportunity for the field of HBI. Across all opportunities, it is vital for HBI researchers to carefully consider the source of data that informs automated decision-support systems to reflect the knowledge, opinions, and needs of broad, diverse communities to ensure that outcomes promote equity and inclusion.

#### Privacy and security

Privacy is a core component of any socio-technical system that collects personal data from its users, and the security of that information is vital, particularly when simultaneously incorporating and sharing information across multiple data sources. Physical spaces in which humans engage have inherent privacy and security concerns related to exchanging information (e.g., meetings) and other sensitive activities (e.g., physical examinations in healthcare settings). As we move towards more intelligent, aware, and adaptive buildings, it is critical to responsibly manage captured data, especially highly personal and potentially sensitive, stigmatic, and exploitable health-associated data. One current example is the rapidly expanding number of distributed IoT devices monitoring building applications (e.g., indoor environmental quality^23, 29, 78, 115^) that are connected to cloud services. This increase in cloud computing is paralleled by an increase in security vulnerabilities and concerns of inappropriate use of data obtained by sensing devices. For example, managers gaining access to cloud-sourced behavioral data of their employees as a means for monitoring performance could lead to employment termination. HBI research should seek to unify and extend current research on security- and privacy-related issues such as smart grid security^116^ and IoT security^117^. Differential privacy^118^ and federated learning^119^ are technical solutions that may be viable solutions to accessing aggregated individual data that prevent access by entities with power over the individuals. Furthermore, increased use of human-centered, participatory design, and generative techniques^120^ are promising ways to advance the security and privacy aspects of HBI by identifying user concerns and accounting for individual preferences. HBI research should consider providing personal user control over privacy settings, identifying policy solutions, and developing new instruments for appropriate data stewardship, such as data trusts^121^. Timely incorporation of privacy and security as considerations in HBI research can ensure effective sharing of data across third-party building applications and between buildings on different networks while maintaining the trust, safety, and security of occupants.

#### Sustainability and resilience

HBI research has an opportunity to move beyond satisfying basic requirements of shelter, safety, and security toward serving broader societal needs such as environmental sustainability, climate resilience, and societal well-being. Building operations are one of the largest sources of global greenhouse gas emissions, with heating, cooling, and water heating comprising most energy-related emissions, particularly in existing built environments. Resource consumption and emissions from built environments are strongly determined by occupant service requirements (temperature, lighting, etc.) and usage patterns. Therefore, HBI research must leverage a deep understanding of these service needs and usage patterns to develop fundamentally sustainable and adaptive design and operation solutions for the built environment that substantially mitigate the risks of catastrophic climate change. The built environment also serves as a primary means of adaptation to the climate crisis and its potential effects on personal comfort, health, and happiness. Even as we work to prevent the disastrous effects of climate change, resilience to these unavoidable events is a must. These impacts often unequally affect communities of color and low-income, emphasizing the necessity for equitable and proactive resilience planning and resource allocation^122^. For instance, the city of Phoenix in Arizona, has developed a heat action plan that relies on natural cooling techniques to cool the neighborhoods most in need^123^. In another example, the city of New Orleans is prone to severe storm events that include hurricanes and flood waters. Therefore, the local authorities issued guidelines for storm preparedness and resilience that include regular maintenance and basic structural improvements strategies^124^. To that end, proponents of the field of HBI should be not only the stewards of our environment but also human health and well-being. Technology's harmful and unintended impacts on human psychology should be carefully considered. This is even more important with the blurring lines of work and home life with the increase in hybrid work^125, 126^; thus, there needs to be accountability for human well-being, safety, comfort, and productivity in HBI-enabled spaces, and HBI innovations should start with a human-centered purpose to influence the development of technology that leads to sustainable and resilient societies.

## Research and practice in HBI

HBI researchers conduct studies in several high-level areas, including (1) discovering and quantifying the passive interaction that impacts users’ personal experiences and outcomes from the built environment and building systems, (2) observing and learning human behaviors in built environments to quantify the active actions and their impact on the built environment and building systems, as well as potentials for technology adoption and success, and (3) designing, building, and evaluating new technology that allows built environments to become more flexible and more responsive to human needs over their operational lifetime. This section provides an overview of methods and practical applications for HBI research. We then present examples of original research in HBI that reflect the different transversal themes outlined above.

### HBI research methods

Research approaches are adopted or adapted from the multiple disciplines and research fields that compose the interdisciplinary field of HBI. Design, development, testing, and user experience research within HBI employs both qualitative (e.g., focus groups, interviews, ethnographies, narratives)^127^ and quantitative (e.g., randomized control trials, experimental studies, observational measurement, surveys) research methods. A variety of AI techniques, optimization methods, including supervised learning (regression, classification, deep learning), unsupervised learning (dimensionality reduction, clustering, anomaly/event detection), and reinforcement learning techniques, are used for predictive modeling, inference, and control. HBI research is conducted in lab-controlled environments^30^, through field studies in existing built environments^15^ and systems^128^, and by the use of immersive virtual reality environments (virtual prototyping)^129^. Simulation is frequently used to understand the impact of HBI technologies and evaluate algorithmic frameworks when human data is unavailable or to develop mathematical models that can be tested against human data/observations^65, 94^.

### Examples of existing HBI applications

Multiple examples of robust HBI applications exist. For example, the Cortellucci Vaughan Hospital in Ontario, Canada, opened in 2021, uses real-time locating systems to track assets and monitor patients’ locations and movements. The hospital also relies on an integrated smart technology grid to maximize information exchange, allowing for efficient logistics management, medical staff workflow, and a pleasant patient experience^130^. The EDGE building in Amsterdam, Holland, is considered one of the most intelligent buildings in the world according to the British rating agency BREEAM^131^. The commercial office building was established to make its users’ work experience as smooth as possible. The building knows workers’ schedules, assigns the most convenient parking spot upon arrival, and customizes the light and temperature of their workstations according to personal preferences^132^. Some Target retail stores in the United States have advanced LED light fixtures with visible light communication (VLC) capabilities. When partnered with Visible Light Positioning (VLP)^133^ of image sensors on smartphones, these LEDs allow customers to use the Target app to navigate around the store to a specific product location^134, 135^. Smart residential buildings equipped with various smart home gadgets (e.g., smart video doorbells, stove controller, smart pillbox, automated emergency call system) provide a comfortable, safe, and secure space, allowing seniors to remain in their homes^136^. Recently, big tech companies have created smart home hubs (e.g., Amazon Echo, Google Home, and Samsung SmartThings), which act as a central house monitor controlling all devices at home and creating a more comfortable living environment for seniors^137^. Finally, during the recent COVID-19 pandemic, the Dubai airport installed smart gates that use the traveler’s face and iris biometrics to reduce passport control procedures and increase security. Airport officers were not required anymore to scan, examine, and check the passports of every traveler, which reduced the human–human interaction during the pandemic and promoted effortless travel^138^.

### Examples of novel applications, proof-of-concepts, and test cases of HBI

As the examples above demonstrate, HBI-centered applications have achieved a maturity level that supports the implementation of automation and service delivery through increasingly available commercial products. State-of-the-art research is being conducted to push the envelope and contribute to more advanced use cases. Examples of research across selected areas of the HBI framework follow.

#### Research example in human experiences, performance, and well-being

**Figure 2:**
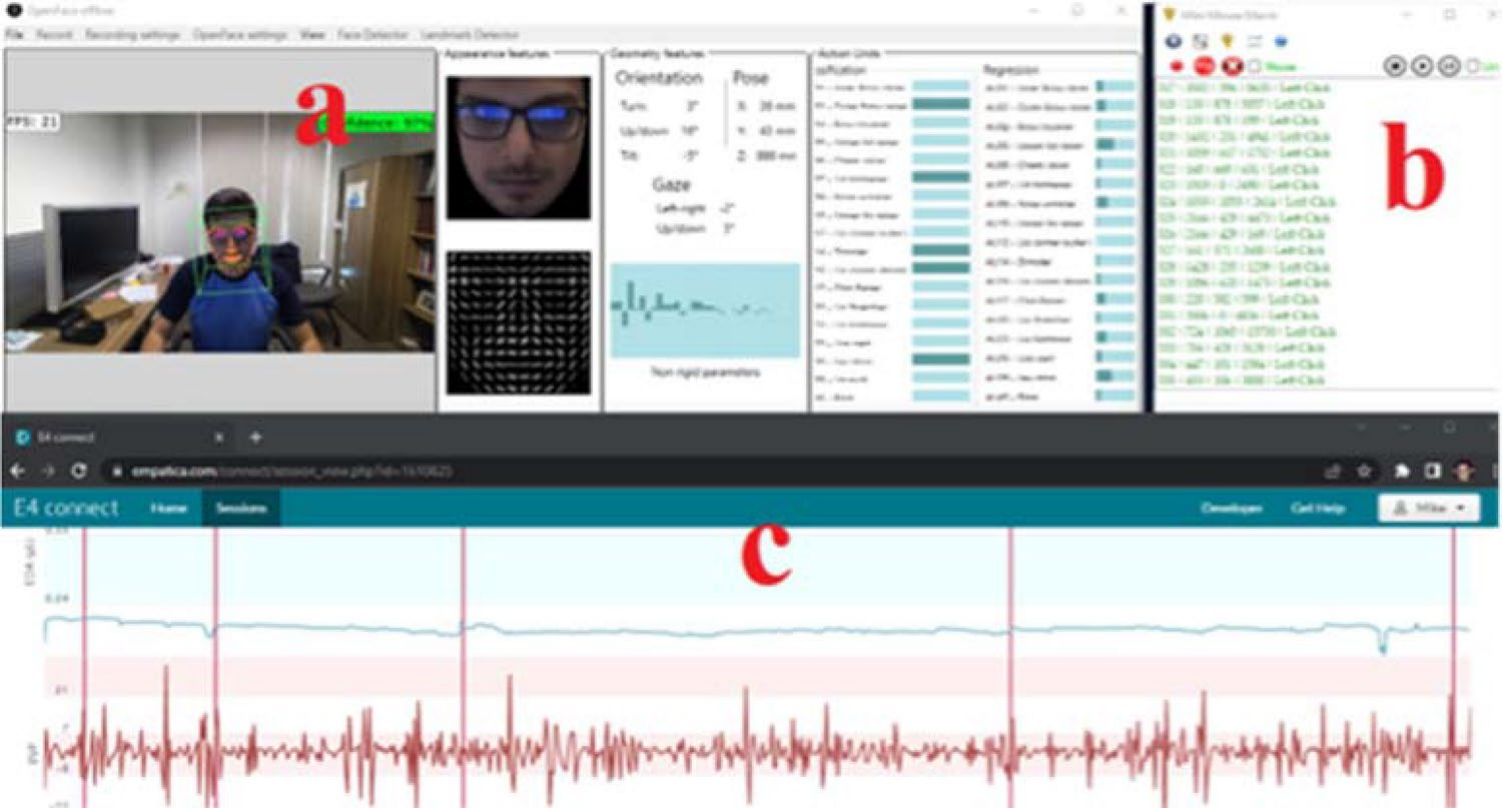
(**a**) Openface application for real-time facial features extraction, (**b**) Human–computer interaction monitoring application, (**c**) real-time physiological data monitoring.

With the outbreak of the SARS-CoV-2 virus, stress levels have increased across the globe, to the extent that building professionals consider stress among the most important mental well-being issues that need to be the focus of design, construction, and operation of buildings^139^. While there are many reasons for stress, one of the top stressors is work pressure. Research suggests that when workers have low control over their job demands (e.g., unfamiliarity with task, limited resources, degraded workspace conditions, etc.) they develop negative stress named distress, which have detrimental psychological and physical health consequences and leads to degraded productivity. On the other hand, when workers feel confident about handling their work stressors, they develop a feeling of eustress known as the positive stress, responsible for motivating people to pursue their goals and to face challenges. Thus, differentiating between eustress and distress is a necessity for work organizations to promote eustress and limit distress among their workers. HBI plays a key role in this innovative research; for example, integrating the appropriate sensors (e.g., wearable sensors, cameras, etc.) into an office workstation sets the foundation for automated systems that rely on physiological and behavioral data to identify whether a worker is witnessing eustress or distress. This helps work mangers shape a strategic plan to assign workers with the appropriate tasks thus sustaining eustress and proactively eliminating distress. In a recent research effort, physiological, behavioral, and human–computer interaction data from 50 office workers have been collected and used to differentiate distress from eustress using machine learning methods (Fig. 2). Furthermore, nature contact has been shown to reduce distress among office workers, therefore the HBI research about stress must investigate the appropriate approaches to deliver nature related interventions to restore distressed office workers to a relaxed state. Finally, unpleasant indoor environmental quality and office interior design have been found to increase workers’ physiological stress, therefore HBI researchers should study the means to create personalized responsive office conditions that can change its ambiance driven by the physiological state of workers.

#### Research example in building design and operations

**Figure 3:**
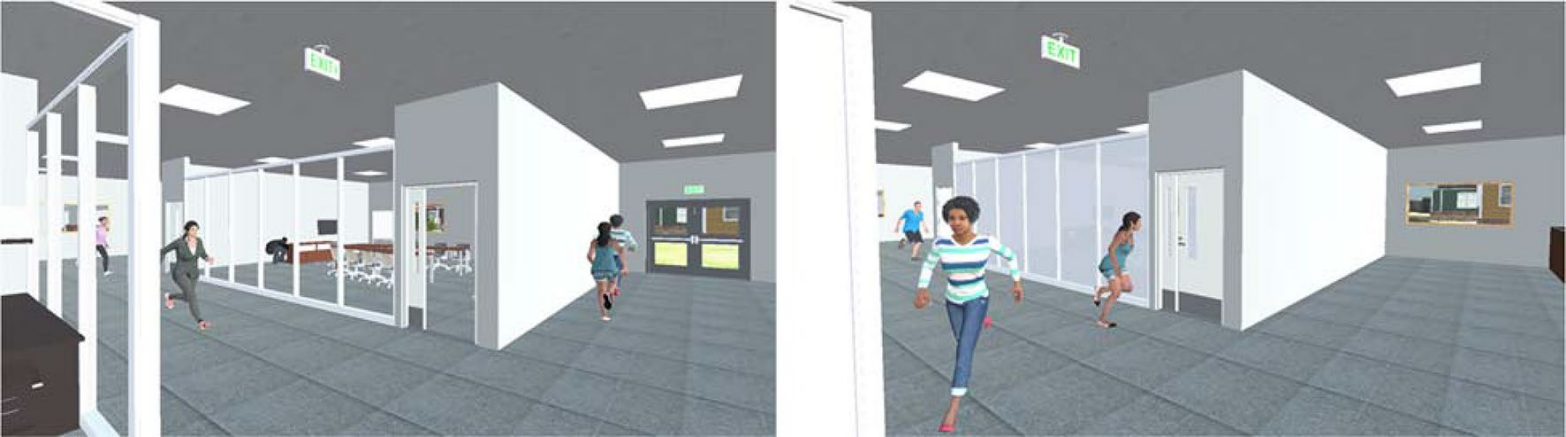
Empirical assessment of the impact of security countermeasures on human behavior during active shooter incidents. The images are from the participants' perspective in a virtual reality-based experiment. Frosted glasses and access control are implemented in the right image. Non-player characters are included to represent social influence during building emergencies.

Building design can impact the behaviors of building occupants. For example, security countermeasures in a building have influenced participants’ response time and decisions in an active shooter incident where participants responded to the emergency in virtual offices and schools^70^ (Fig. 3). The study also revealed the importance of building and social context such that teachers concerned more for others’ safety than office workers^70^. These explorations reveal the necessity of customizing building design and operations to provide resilient solutions to extreme events for different users, which can be accomplished using virtual reality. Given its sufficient ecological validity and flexibility, virtual reality can also be an ideal platform to train building occupants on emergency safety, where trainees immerse into the scenarios and interact with the environment that can be customized per needs (e.g., office, school, hospitals). Similarly, the training outcomes will then be tested in a virtual emergency scenario where people’s performance in the environment is evaluated by customizable systematic metrics developed based on training contents (e.g., evacuation time and execution of safety actions).

#### Research example in sensing, inference, and awareness

**Figure 4:**
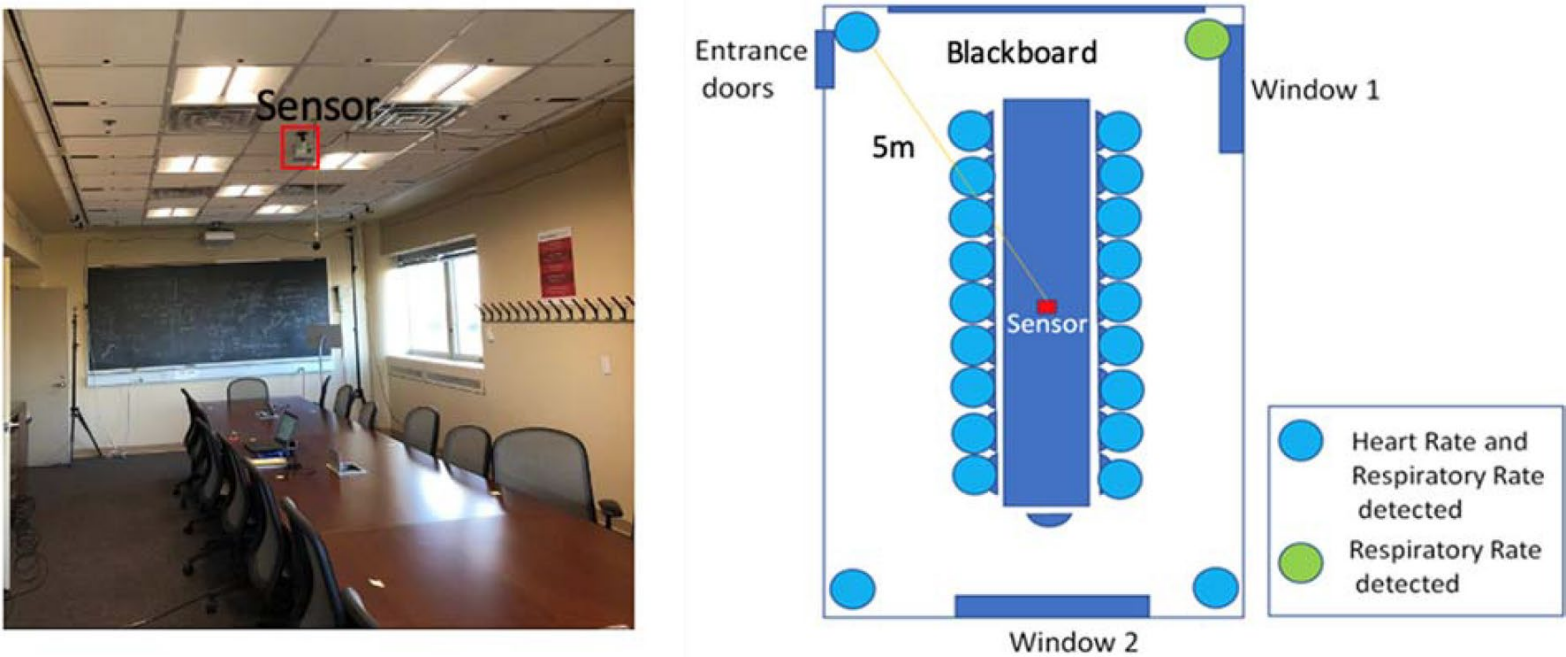
The 3.4 m × 8.5 m LESA Smart Conference Room with the TruePODS™ sensor mounted on the ceiling (left), and locations of successful physiological detection (right). Heart rates were detected at all the seats around the table, and in 3 of the 4 corners. In all locations, respiratory rate was detected.

Doppler radar physiological sensing using dedicated radar systems and wireless infrastructure-based systems has been shown to be effective in sensing physiological parameters from several meters to several tens of meters with a high degree of accuracy in controlled settings^32^. Physiological parameters include heart and respiratory rates, respiratory tidal volume, heart rate variability (HRV), pulse pressure, diagnostic patterns, activity level, and body orientation. These parameters can be used to assess user response to environmental conditions. One example is True Presence Occupancy Detection Sensor (TruePODS™), which was developed by a start-up company Adnoviv, Inc. in collaboration with the University of Hawaii. TruePODS™ is a Doppler radar-based occupancy sensor that detects breathing to accurately indicate whether a space is occupied, even when the occupant is sedentary. TruePODS™ modules have been tested at Rensselaer Polytechnic Institute LESA (Light Enabled Systems & Applications) Center, focused on using light efficiently in built environments, and healthcare, among others. The LESA smart conference room (Fig. 4) senses occupancy, pose (sitting, falling, standing), and has a mesh network of color sensors for coarse occupancy sensing and measuring reflected sunlight and solar heat flux for improved HVAC control. The TruePODS™ module has been validated for occupancy/vacancy detection and occupant count and can successfully measure respiratory and heart rate when used in certain positions and orientations (Fig. 4). This is the first demonstration of an occupancy/vacancy sensor that also provides occupant count and physiological parameters. The sensor output may enable more energy-efficient building control while ensuring that occupant comfort is not compromised.

#### Research example in trust and collaboration

**Figure 5:**
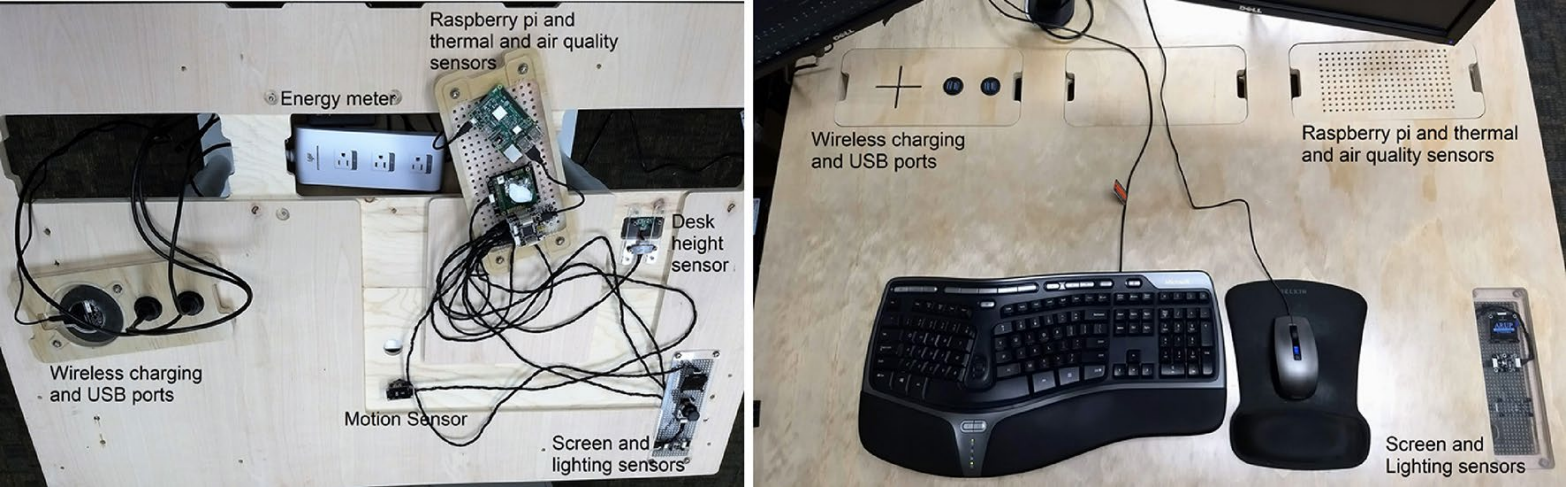
Intelligent workstation with embedded sensors to learn and adapt indoor environment based on user preferences.

Centralized environmental controls in built environments are frequently unable to meet the needs of most occupants^140^. Alternatively, automated control of local environmental factors (e.g., thermal, lighting) and equipment positioning (e.g., a motorized sit/stand desk^141^) shows promise in supporting worker health and well-being. In one study, multiple examples of trust and collaboration are emerging in the development of an intelligent office workstation to address these concerns^19^. Facilitated by learning from interactions between an intelligent workstation (Fig. 5) and the occupant, an intelligent office workstation would understand and adapt to the worker’s changing needs in terms of thermal, lighting, and posture comfort, share control with the user to build trust, and will coevolve with its user to promote healthier workplace behavior (A short video describing this research can be found at: https://youtu.be/psfzIDTgK5g). That is, the smart desk and user collaborate to find and use the best settings for important outcomes in addition to comfort, including health and productivity. A real-world evaluation showed that sharing the control between the smart desk and the user to control the thermal environment led to higher satisfaction than in manually controlled environments^20^, opening an avenue for collaborative control and trust between a building and its occupants. In addition to collaboration in making changes, a user-centered approach using focus groups has highlighted numerous trust considerations linked to the privacy and security of the data collected by the workstation^142^.

#### Research example in modeling and simulation

**Figure 6:**
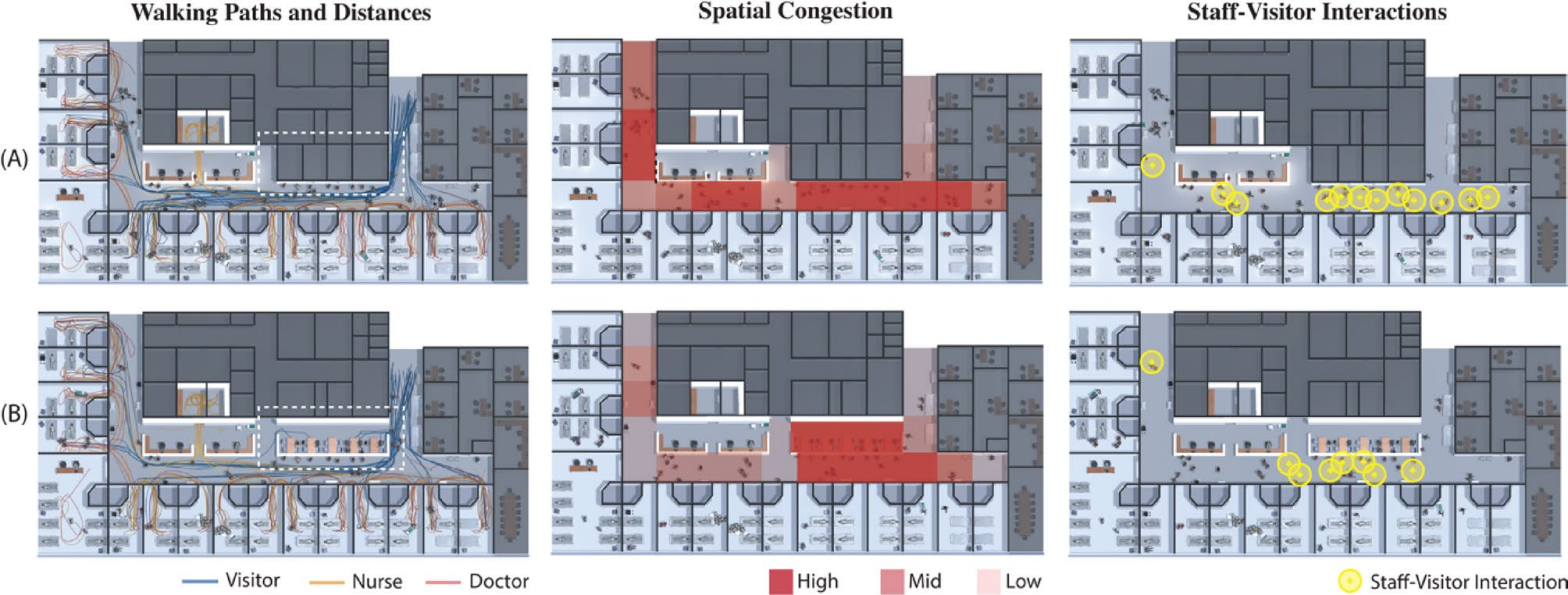
Comparative evaluation of the impact of two design strategies on the inhabitants' behavior. The design difference relates to the presence (Design A) or absence (Design B) of a dayroom (marked with a white dotted line on the floorplan) where patients and visitors can gather to engage in social interactions. Results indicate that the presence of a dayroom reduces people density in corridors and leads to fewer interruptions for the medical staff involved in patient care activities. Figure adapted from^143^.

Pre-occupancy analyses are a valuable tool for architects and other stakeholders to forecast how a proposed design will affect the behavior of a building's future inhabitants. Their goal is to augment the knowledge and intuition of architects to reduce the gap between the expected and actual performance of a built environment. Among them, multi-agent simulation approaches represent the dynamic interactions between occupants and a physical environment. A recent study used a narrative-based approach to predict and evaluate the impact of two alternative design configurations for an internal medicine ward on the day-to-day behavior of the occupants (e.g., doctors, nurses, patients, visitors)^143^. A combination of field observations and expert interviews informed the modeling of a digital ‘place,’ composed of spaces, actors, activities, and narrative models where synthetic people (modeled as agents) inhabit a virtual space and perform a set of individual or collaborative activities consistent with the function of the organization that occupies the built environment. The space model includes physical and non-physical attributes, which determine the range of behaviors that can be hosted at a given time contingent upon physical (e.g., geometry) and social (e.g., presence and activities of other occupants in the same space) contingencies. Actor models include specific occupant roles and dynamic attributes (e.g., walked distances, time spent in specific activities). The activity model determines interactions between spaces and occupants (e.g., moving, queuing, interacting), while the narrative model assigns actors to one or more spaces to achieve a goal-oriented task. This simulation approach distributes intelligence not only in agents but also in spaces, activity, and narrative models to ease the process of modeling complex and collaborative behaviors. The simulation results shown in Fig. 6 reveal the implications of two alternative designs for an outpatient clinic on people’s travel paths, occupancy density, and frequency of staff-visitor interactions. Specifically, the presence of a dayroom reduces visitors’ density in corridors, which could cause spatial bottlenecks, and it diminishes the number of unplanned staff–visitor interactions that can delay the performance of routine medical procedures.

#### Research example in equity and inclusion

**Figure 7:**
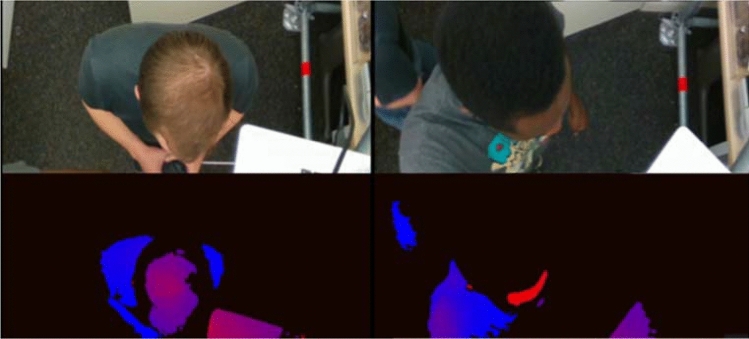
Color (top) and depth (bottom) image of two human subjects with different hair types as measured by an active infrared stereo camera. The depth map is color-coded such that darker (more black) is farther away from the camera while lighter (more red) is closer.

A new class of occupancy estimation sensing based on active infrared stereo technologies obtains a depth image from a sensor located in a doorway and uses this image to detect entrance and exit events^11^. Though much more accurate than traditional passive-infrared (PIR) sensors at detecting and estimating occupancy, the configuration of the sensing solution is such that the entrance and exit events of certain occupants are misrepresented in the output. In particular, the depth-imaging system relies on infrared light, which is absorbed differently by different hair types. Since the software processing the images is tracking heads as they move through the doorway, some hair types create a pattern that confuses the algorithm that oversees detecting and tracking heads leading to a misestimation of the occupancy in the room. Figure 7 shows a sample depth map obtained from the sensor with two subjects with different hair types. This example shows the importance of developing and selecting sensors that do not result in biased results. Similarly, algorithm bias is a well-acknowledged issue due to erroneous assumptions in machine learning process and training on biased data sets.

## A path forward

As an emerging field that incorporates the dynamic interplay of human experience and building intelligence, the primary aim of this paper was to specify the definition, vision, and research dimensions of HBI. Toward that goal, this paper unpacked three primary areas that contribute to and require attention in HBI research: humans (human experiences, performance, and well-being), buildings (building design and operations), and technology (sensing, inference, and awareness). We have presented the critical interdisciplinary research domains at the intersections of these three primary areas: trust and collaboration, decision-making and control, and modeling and simulation. Finally, we have described core principles for all HBI research to consider and address, including equity, privacy, and sustainability. Across the framework, we provide *questions* meant to stimulate collaborative and widespread HBI research efforts. Similarly, we have presented examples of existing HBI applications and emerging original research to inspire individuals interested in advancing HBI research and application. Taken together, this information is meant to support HBI researchers, designers, and practitioners in considering the wide range of symbiotic and interactive possibilities for humans and the built environment. Ultimately, promoting thoughtful interdisciplinary approaches to HBI research will encourage the development of systems sensitive to the needs of occupants while maximizing the operational goals of built environments, resulting in inclusive, safe, and exciting places for people to live, work, and play.

## Data Availability

The data that support the findings of this study are available from the authors, but restrictions apply to the availability of these data, which were used under license for the current study, and so are not publicly available. Data are however available from the corresponding author upon reasonable request and with permission of the respective universities (USC, CMU, University of Technion, University of Hawaii).
